# Comparison between Transepithelial Photorefractive Keratectomy versus Alcohol-Assisted Photorefractive Keratectomy in Correction of Myopia and Myopic Astigmatism

**DOI:** 10.1155/2018/5376235

**Published:** 2018-11-12

**Authors:** Ashraf M. Bakhsh, Shaaban A. M. Elwan, Ahsan A. Chaudhry, Tamer M. El-Atris, Taghleb M. Al-Howish

**Affiliations:** ^1^Ophthalmology Department, Security Forces Hospital, Riyadh, Saudi Arabia; ^2^Ophthalmology Department, Faculty of Medicine, Alfaisal University, Riyadh, Saudi Arabia; ^3^Ophthalmology Department, Faculty of Medicine, Minia University, El-Minia, Egypt

## Abstract

**Purpose:**

The aim of the study was to compare visual acuity, refractive results, safety, and efficacy of TPRK with AAPRK as primary outcomes and surgical time, pain scores, haze levels, and healing time as secondary outcomes in TPRK and AAPRK groups.

**Setting:**

Security Forces Hospital, Ophthalmology Department, Riyadh, Kingdom of Saudi Arabia.

**Design:**

Prospective, nonrandomized case-control comparative study.

**Methods:**

A total of 200 eyes of 100 consecutive patients were included. One hundred eyes underwent TPRK in the right eye (study group), and 100 eyes underwent AAPRK in the left eye (control group). Ablations were performed with the Schwind Amaris excimer LASER750S. Clinical outcomes during 6 months' follow-up were compared.

**Results:**

The mean age of patients was 28.3 ± 6.3, 77 were females and 23 males. The mean surgical time was 162.17 ±  14.827 s and 243.24  ±  98.69 s, respectively. At day 1, the UDVA mean was 0.7 in 87% of eyes in the TPRK group while it was 0.5 in 45% of eyes in AAPRK; at week 1, it was 0.9 in 88% of eyes in the TPRK group and 0.6 in 60% of eyes in AAPRK. The mean pain scores were less and lower incidence of corneal haze in the TPRK. Complete epithelial healing time was shorter in TPRK, 3.20 ± 0.686 and 4.60 ± 1.969 days, respectively.

**Conclusions:**

TPRK and AAPRK produce similar results 6 months postoperatively. However, in the early postoperative period, there were significant differences in UDVA, pain score, level of haze, and complete epithelial healing time. The pain scores were lower, level of haze was less, and healing time was shorter in the TPRK group which provided patient better felling and comfort in this period. Both of procedures are effective and safe for correction of myopia and compound myopic astigmatism. This trial is registered with NCT03569423.

## 1. Introduction

Previous studies document that, although Laser in situ keratomileusis (LASIK) is the most worldwide performed refractive procedure, surface ablation may be safer to avoid flap complications, corneal weakening, and a higher risk of iatrogenic keratectasia associated with LASIK, and thus, the era of surface ablation emerged as an alternative [1–3]. Photorefractive keratectomy (PRK) is one of the surface ablation procedures and performed after corneal epithelial debridement accompanied by postoperative pain, discomfort, and high grade of corneal haze, all of which limit its popularity [4]. The traditional method for corneal epithelium removal before excimer LASER was manual scraping, which was later enhanced by using an alcohol solution 20% or brush [5]. In 2003, Camellin [6] introduced a new alcohol-assisted technique called laser-assisted subepithelial keratectomy (LASEK) that allowed the epithelium to be preserved as a flap and applied back to the stromal corneal bed after laser treatment. Also in 2003, Pallikaris et al. [7] invented epithelial laser in situ keratomileusis (epi-LASIK) which is another method that uses the epithelial flap and performed with a microkeratome (called epi-keratome) with a blunt oscillating blade. After transepithelial photorefractive keratectomy (TPRK) was introduced, removal of the epithelium is done by phototherapeutic ablation followed by refractive ablation of the corneal stroma. Several studies emerged and advocated many techniques for epithelial removal, but this 2-step technique was not worldwide used due to the prolonged surgery time with the older generation of lasers, corneal dehydration, increased postoperative pain, and a deficiency of adjusted nomograms [8–11]. When new generations of faster lasers, improved ablation algorithms, and nomograms have emerged, it allowed the development of a new TPRK nontouch: all-surface ablation technique which allows ablation of the corneal epithelium and stroma in a single step with one ablation profile. This aspheric profile is calculated according to data from the literature, estimating that the normal corneal epithelial thickness is 55 *μ*m centrally and 65 peripherally at 4 mm radially from the center [12]. A number of recent studies demonstrated that this single-step TPRK is a relatively new procedure with many advantages such as reduced surgical time, minimizing the size of epithelial defect to that required for stromal ablation, no alcohol use avoiding potential toxicity to the limbal cells, less postoperative pain, and corneal haze with rapid healing time and faster visual recovery [13–16]. Thus, there is a need for an updated comparative evaluation based on a larger number of eyes. The aim of our study was to compare 6-month uncorrected distant visual acuity (UDVA) and best corrected distant visual acuity (BCDVA) means, refractive results, safety, and efficacy of single-step TPRK with alcohol-assisted PRK (AAPRK) as primary outcomes and comparison of surgical time, pain scores, and haze levels, complete epithelial healing time, as secondary outcomes among the two procedures when used to correct mild to moderate myopia and myopic astigmatism.

### 1.1. Subjects and Methods

Our study is a prospective, consecutive, and nonrandomized cohort study that includes eyes that underwent either single-step TPRK or AAPRK between February 2017 and April 2018, at the Security Forces Hospital, Ophthalmology Department, Riyadh, Kingdom of Saudi Arabia. The study was approved by the local ethical board committee. Before the surgical procedure, each patient was adequately informed about the study as well as the risks and benefits of the surgery and signed informed consent in accordance with the Declaration of Helsinki. The inclusion criteria were as follows: age over 18 years, primary myopia or compound myopic astigmatism, preoperative manifest refraction spherical equivalent (MRSE) within the range of −1.50 to −7 *D*, a stable refraction for at least 1 year before the surgery, contact lens discontinuation for at least 3 weeks, and an estimated stromal corneal bed thickness of >330 *μ*m at the thinnest location. Exclusion criteria were previous ocular surgery, active ocular diseases, corneal dystrophy, retinal disease, glaucoma, dry eye, a history of severe eye trauma, irregular astigmatism or suspected keratoconus on corneal topography, and systemic disease that could affect corneal wound healing such as collagen diseases, diabetes mellitus, and pregnancy.

A total of 200 eyes of 100 consecutive patients were included; one hundred eyes underwent TPRK (study group) and 100 eyes underwent AAPRK (control group). The study design choice of the procedure was fixed for each patient: the right eye underwent TPRK and the contralateral left eye AAPRK. Patient's demographics and preoperative variables are demonstrated in Table 1. There are no significant differences in preoperative variables of patients in the TPRK and AAPRK groups. The percentage of females is 77% and males 23%. Patients who attended all visits, without any missing data, were included in the statistical analysis.

#### 1.1.1. Preoperative Examination

The preoperative examination included UDVA, BCDVA, manifest and cycloplegic refraction, slit lamp biomicroscopy, tonometry, Pentacam camera (OCULUS-Netzteil Art., Pentacam HR, Germany), tomography (Sirius, SCHWIND eye-tech-solutions GmbH, Kleinostheim, Germany), and dilated fundus examination using binocular ophthalmoscopy. Contact lenses use and medical history, including any systemic diseases, were recorded.

#### 1.1.2. Surgical Technique

All surgeries were performed with 6th-generation Amaris excimer LASER 193 nm, version 750 S (Schwind eye-tech-solutions GmbH & Co.,KG, Mainparkstrasse, Kleinostheim, Germany). Ablations were based on aberration-free algorithms calculated using ORK-CAM software with the beam size of 0.54 mm full width and high-speed eye tracking. Treatments were performed by 2 surgeons (BA and ESH) using an identical surgical protocol. The treatments were mostly aimed at emmetropia. Before the surgery, tetracaine hydrochloride 0.5% ophthalmic solution (Bausch & Lomb, Minims) and moxifloxacin 0.5% (Vigamox, Alcon Co.) drops were instilled 3 times within a 5-minute interval. The eyelids were prepared with antiseptic chlorhexidine gluconate 0.05% solution (Saudi Medical Solution Company) and opened using a wire lid speculum. In the AAPRK group, the cornea was exposed to a 20% ethyl alcohol solution for 25 seconds with the use of a well. Subsequently, a superficial cut of the epithelium was made with either an 8.5 or 9.5 mm diameter trephine. The epithelium was mechanically debrided with the well or with a blunt spatula, and then, LASER treatment with the same machine was initiated. In the TPRK group, where aspheric aberration-free TPRK ablation algorithm was used (Schwind eye-tech-solutions), the epithelium was removed during laser ablation only from the area of the total ablation zone. In both groups and in all cases, immediately after treatment, the eye was washed with balanced salt solution (BSS) for 20 seconds. Then, to fight against postoperative corneal haze, mitomycin C (MMC) 0.02% was applied for 30 seconds followed by copious irrigation of the eye with BSS. Intraoperative complications were not noted, and surgical time starting from eyelid speculum insertion to the time of its removal at the end of the procedure was recorded. After the surgery, a bandage contact lens was applied (BIOMEDICS Evolution CL ocufilcon *D* 45%, water 55%) for 7 days. The postoperative regimen included tobradex eye drops 0.3% (tobramycin 0.3%-dexamethasone 0.1% sterile eye drops, Alcon Co.) with tapering dose for 1 month starting with QID/1 week, TID/1 week, BID/1 week and once a day/1 week, moxifloxacin drops 0.5% (Vigamox, Alcon Co.) for 2 weeks, and sodium hyaluronate 0.2% (Hyfresh eye drops, Jamjoom Pharma Co.) drop/2 hours and a gradual decreasing of the frequency for 3 months. A pain killer oral medication tablet/6 hours was used in the first postoperative days if needed.

#### 1.1.3. Postoperative Examinations

Patients were instructed to visit the clinic for postoperative examinations and follow-up after 1 day, 1 week, 1 month, 3 months, and 6 months. The observers were unmasked, but the patients were not told which eye had either TPRK or AAPRK surgery. Examinations at 1 day, 1 week, 1 month, 3 months, and 6 months included UDVA, but the BCDVA and manifest refraction were measured at 1, 3, and 6 months. Slit lamp biomicroscopy was done in each visit. Corneal haze grading was evaluated according to Fantes et al.'s [17] proposal (0  =  no haze; 0.5  =  trace haze on oblique illumination; 1  =  corneal cloudiness not interfering with the visibility of fine iris details; 2  =  mild effacement of fine iris details; 3 and 4  =  details of the lens and iris not discernible). Healing time in which complete reepithelialisation occurred in both eyes was recorded. In postoperative day 1, day 3, and week 1, we used a discrete, 11-category numeric pain scale (NPS, 0 = no pain and 10 = the worst possible pain) to evaluate pain score in each eye, and patients response were recorded at the early postoperative period. Six months postoperatively, patients were asked about the overall satisfaction with each procedure as high, moderate, low, and not satisfied, and whether they would decide to have the surgery again (yes, no) was recorded.

#### 1.1.4. Statistical Analysis

Patients' data were entered in Microsoft Excel, copied, and analyzed using SigmaPlot-Scientific Data Program for the 2 groups, and paired Student's *t*-test was used for the UDVA and BCDVA means in decimal values and for MRSE means. The Mann–Whitney *U* test was used for pain scores, haze levels, and healing time. For all tests, a *P* value <  0.05 was considered statistically significant. A Graph Pad Prism 5 program was used for graphs constructions.

## 2. Results

**Figure 1:**
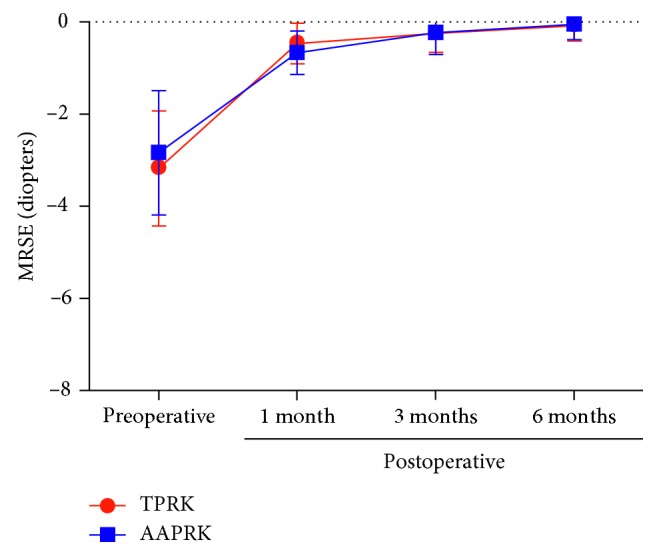
Manifest refraction spherical equivalent (MRSE) in TPRK and AAPRK groups. Data are expressed as means ± SD.

The mean age of the patients was 28.3 ± 6.3 years (range 18–50 years); 77 were females and 23 were males. Table 1 shows the demographic data for TPRK and AAPRK groups, respectively, in which the mean preoperative MRSE was −3.158 ± 1.596 and −2.90 ± 1.899 *D* (*P*=0.089), and the refractive astigmatism is less than −1.50 *D* in both groups. The mean keratometry reading was 44.50 ± 1.45 and 43.60 ± 1.70 d (*P*=0.33), the mean preoperative central corneal thickness (CCT) was 490 ± 4.80 and 496 ± 4.60 *μ*m (*P*=0.308), the minimal estimated stromal residual thickness was 340 ± 18 in the TPRK group and 345 ± 17 *μ*m (*P*=0.33) in the AAPRK group, the mean ablation time was 29.71  ±  7.62 s in the TPRK group and 12.33  ±  6.138 s in the AAPRK group (*P* < 0.001), whereas the mean surgical time of the whole procedure was 162.17 ± 14.827 s and 243.24  ±  98.69 s, respectively (*P* < 0.001). In the TPRK and AAPRK groups, respectively, the mean diameter of the optical zone was 6.734  ±  0.194 and 6.745  ±  0.201 mm (*P*=0.988) and the transition zone was 1.036 ± 0.371 and 0.995 ± 0.364 mm (*P*=0.645). There were no statistically significant differences between the two groups regarding the mean 1, 3, and 6 months postoperative MRSE, as shown in Figure 1; the mean postoperative MRSE at 1 month was −0.14 0 ± 0.441 *D* in the TPRK group and −0.165 ±  0.476 *D* in the AAPRK group (*P*=0.986). The mean postoperative MRSE at 3 months was −0.110 ± 0.418 *D* in the TPRK group and −0.113 ± 0.418 in the AAPRK group (*P*=1). The mean postoperative MRSE at 6 months was −0.0500 ± 0.337 *D* in the TPRK group and −0.0450 ± 0.338 *D* in the AAPRK group (*P*=1).

**Figure 2:**
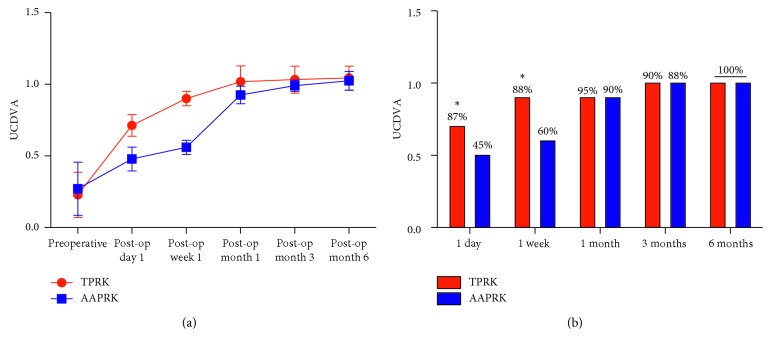
(a) Preoperative and postoperative uncorrected distant visual acuity (UDVA) in decimal values in both groups. (b) The postoperative UDVA percentage of eyes improvement over time in both groups. Data are expressed as means ± SD.

**Figure 3:**
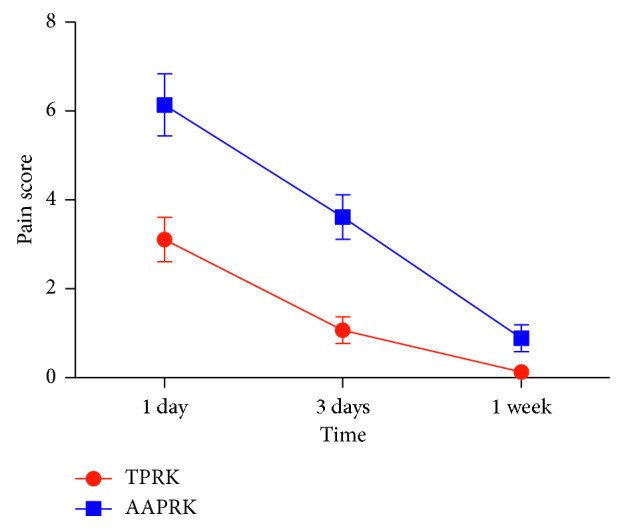
The mean postoperative pain scores in both groups. Data are expressed as means ± SD.

**Figure 4:**
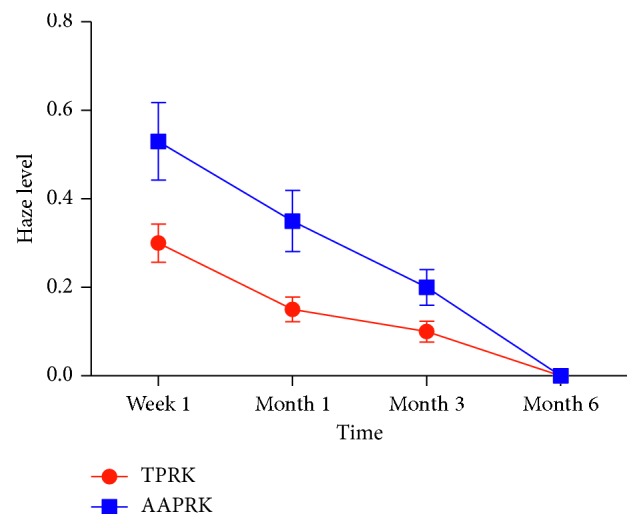
The mean level of haze according to Fantes scores in both groups. Data are expressed as means ± SD.

**Figure 5:**
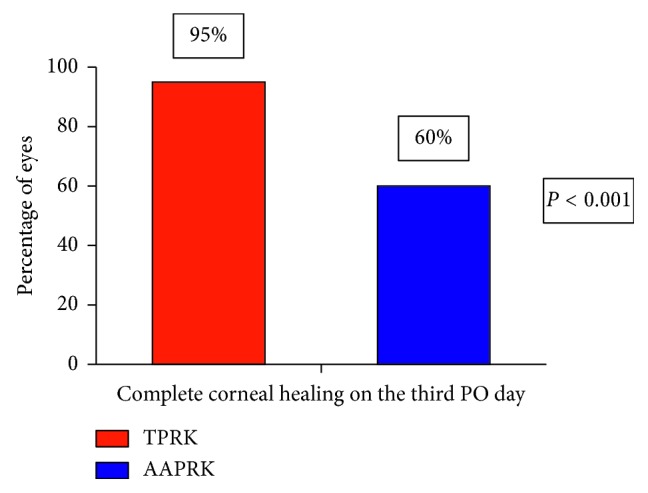
Percentage of eyes with complete corneal epithelial healing on the third postoperative day. Data are expressed as means ± SD.

At 3 months, the postoperative mean MRSE was in 63% of eyes within ± 0.25 *D*, 20% within +0.25 to ± 0.5 *D*, 17% within −0.50 to −0.75 *D*, and no eyes (0%) within −0.75 to −1.0 *D*; the respective values in the AAPRK group were 63%, 21%, 15%, and 1%. The differences were not statistically significant. Regarding the preoperative mean UDVA and BCDVA, there are no statistically significant differences between groups in which it was for UDVA 0.229 ± 0.158 in the TPRK group and 0.272 ± 0.185 in the AAPRK group (*P*=0.981) and for BCDVA 1.001 ± 0.0577 in the TPRK group and 1.006 ± 0.0052 in the AAPRK group (*P*=0.15).  Figure 2(a) shows the postoperative mean UDVA comparing the two groups: there were statistically significant differences at 1 day, 1 week, and 1 month (*P* < 0.001), with visual stability (< ± 0.50 *D* difference in two consecutive visits) as well as no significant differences at 3 and 6 months (*P*=0.081 & 0.613); the values were 0.713 ± 0.074 (range, 0.5–0.9), 0.901 ± 0.050 (range, 0.8–1), 1.018 ± 0.110 (range, 0.8–1.2), 1.033 ± 0.094 (range, 0.9–1.2), and 1.044 ± 0.083 (range, 1–1.2), respectively, in the TPRK group while the values were 0.479 ± 0.083 (range, 0.3–0.6), 0.56 ± 0.049 (range, 0.5–0.6), 0.926 ± 0.061 (range, 0.8–1.), 0.990 ± 0.038 (range, 0.9–1.2), and 1.024 ± 0.065 (range, 1–1.2), respectively, in the AAPRK group.  Figure 2(b) shows the postoperative mean UDVA comparing the two groups; there were statistically significant differences in early postoperative time at day 1 and week 1 (*P* < 0.001), where at day 1, it was 0.7 in 87% of eyes in the TPRK group and 0.5 in 45% of eyes in AAPRK and at week 1, it was 0.9 in 88% of eyes in the TPRK group and 0.6 in 60% of eyes in AAPRK. The postoperative mean UDVA values were 0.9 in 95%, 1 in 90%, and ≥1 in 100% at 1, 3, and 6 months, respectively, in the TPRK group while the corresponding values were 0.9 in 90%, 1 in 88%, and ≥1 in 100% in the AAPRK group. There are no statistically significant differences in postoperative BCDVA mean in both of groups at 1, 3, and 6 months, where the values were 1.027 ± 0.101, 1.036 ± 0.091, and 1.044 ± 0.083 in the TPRK group, respectively, and the corresponding values were 0.936 ± 0.057, 0.993 ± 0.035, and 1.024 ± 0.065 in the AAPRK group, respectively. Regarding the efficacy of the procedures, we compared the ratio of the mean postoperative UDVA to the mean preoperative BCDVA (efficacy index) for both groups, as shown in Table 2. The index was better in TPRK than the AAPRK group at day 1 and week 1, in which the difference was statistically significant (*P* < 0.001), but no difference on the other tested time points was detected. For the safety of the procedure, we compared the ratio of the mean postoperative BCDVA to the mean preoperative BCDVA (safety index) for both groups, as shown in Table 3. There is no significant difference found, as well as no eye lost one or more lines of the preoperative BCDVA in both groups. The mean pain scores after the surgery as shown in Figure 3 at day 1, day 3, and week 1 were recorded as 3.110 ± 1.325, 1.070 ± 1.328, and 0.130 ± 0.580 in the TPRK group, respectively, and the corresponding values in the AAPRK group were 6.140 ± 1.815, 3.620 ± 1.523, and 0.890 ± 0.994 with statistically significant differences in all tested time points, where the *P* values were <0.001. A lower incidence of postoperative corneal haze was detected with the slit lamp for the TPRK group compared to the AAPRK group at all tested time points: 1 week, 1 month, and 3 months, as shown in Figure 4, in which the mean results were 0.300 ± 0.432, 0.150 ± 0.280, and 0.100 ± 0.236 in the TPRK group, respectively, and the corresponding values in the AAPRK group were 0.530 ± 0.876, 0.350 ± 0.687, and 0.200 ± 0.402 with statistically significant differences in all tested time points, where the *P* values were 0.019, 0.008, and 0.033, respectively. During the follow-up, the corneal haze intensity had a tendency to decrease until reaching to postoperative 6 months, where there was no haze in both the groups. Regarding complete corneal epithelial healing time, the mean was shorter in the TPRK group than in AAPRK, in which it was 3.20 ± 0.686 and 4.60 ± 1.969 days, respectively (*P* < 0.001), and as shown in Figure 5, complete healing was obtained in the third postoperative day in 95% of eyes in the TPRK group in contrast to 60% in the AAPRK group (*P* < 0.001).

At the end of the study, in the TPRK group, 90% of patients declared high satisfaction with the surgery compared to 88% of patients in AAPRK (*P*=0.46). The ratio for moderate satisfaction was 10% for TPRK and 12% for AAPRK, respectively (*P*=0.45), and none of patients recorded with low or no satisfaction. All patients would consider having the surgery again. There was no statistically significant difference in the incidence of other postoperative complications, which included more intensive dry eye symptoms in 3% of eyes after TPRK and 4% after AAPRK and decreased visual acuity at night tested by covering one eye alternatively, and it was 2% of eyes after TPRK and 3% after AAPRK. No postoperative complications, such as keratitis, delayed reepithelialization, or recurrent corneal erosion, were reported to a level of clinical significance in our study.

## 3. Discussion

TPRK was known as a complementary procedure after LASIK, keratoplasty, and radial keratotomy [18–20]. In 1998, Clinch et al. [9] considered TPRK as the main treatment option; however, this idea was not proven until 2007. In 2007, Ghadhfan et al. [21] demonstrated better results with TPRK than LASIK, LASEK, and epi-LASIK. Fadlallah et al. [13] provided that TPRK was safer and easier to perform than conventional PRK in treatment of mild to moderate myopia. In our study, preoperative refractive mean values and demographic data among both TPRK and AAPRK groups were similar because we performed TPRK in one eye (right eye) and AAPRK in the contralateral eye (left eye) of the same patients. Surgical time was shorter by 66.6% in TPRK than the AAPRK group and this result is conceded as an advantage of TPRK as it reduces the risk of corneal dehydration and similar results were obtained by Celik et al. [22], in which it was shorter by 60% (58.0 ± 6.4 s in TPRK and 98.6 ± 9.8 s in m-PRK eyes); however, they studied 84 eyes while we studied 200 eyes. Regarding the primary outcomes of visual results, there were no statistically significant differences between the two groups regarding the mean of postoperative MRSE at all-time tested points as well as no significant differences at 3 and 6 months in postoperative mean UDVA, BCDVA, and safety index; however, there were statistically significant differences in the early postoperative period at day 1, week 1, and month 1 in UDVA and in the efficacy index at day 1 and week 1 for the TPRK group as patients' eyes recovered faster and inconsistently at the same time in contrast to those of the AAPRK group; some eyes improved fast whilst others took 2-3 months; those who did not experience a fast improvement were told that they will eventually get better with visual stability after 3 months. These results reflect the safety, efficacy, and predictability of both procedures as well as in agreement with the similar results achieved by Fadlallah et al. [13], Celik et al. [22], and Ghobashy et al. [23]. Moreover, these results were superior to that obtained by Wang et al. [24], in which our value of the mean postoperative UDVA in the TPRK group at 1 month was 0.9 in 95% of eyes compared to 39% in their study because they used an older version of excimer LASER: Schwind ESIRIS two-step mode machine. The study demonstrated decreased mean postoperative pain scores in TPRK than the AAPRK group with statistically significant differences at day 1, day 3, and week 1 (*P* < 0.001) as well as similar results were obtained by Fadlallah et al. [13], in which their postoperative pain score at 48 hours was 2.0 in the TPRK and 4.5 in the AAPRK (*P*=0.02), and also in agreement with Celik et al.'s [22] results, but in disagreement with Kanitkar et al. [10], in which their pain score is less in AAPRK than in the TPRK group. A lower incidence of postoperative corneal haze was detected in the TPRK group compared to the AAPRK group in our study at all-time tested points 1 week, 1 month, and 3 months with statistically significant differences possibly due to less keratocyte loss and apoptosis, no alcohol-induced toxicity, less epithelial injury (nontouch technique), and hence less haze formation in the TPRK group. This finding is similar to that of Helena et al. [25], in which they reported quantitative and qualitative differences in keratocyte apoptosis among LASIK, epithelial scrape-PRK and TPRK, and similar to that of Celik et al. [22], but in contrary with an old study of Muller-Pedersen et al. [26], in which they reported increased keratocyte activation, intense inflammatory response, and mylofibroblast transformation in TPRK. However, during the follow-up corneal haze intensity had a tendency to decrease until reaching to postoperative 6 months where there was no haze detected; however, unlike their scheme, we used MMC 0.02% in both groups. Complete corneal epithelial healing time was shorter in TPRK than the AAPRK group in our study; this was probably due to the uniform, precise, and smooth epithelial treatment, and the area removed was the same as that of the treated zone in the TPRK, in contrast to the area removed in AAPRK, where it was larger than the treated zone by LASER. Similar results were obtained by Lee et al. [11], in which they reported that LASER epithelial removal is the shortest in healing time among the three epithelial removal techniques: mechanical, alcohol-assisted, and excimer LASER, as well as similar results with Fadlallah et al. [13], in which they reported 2.5 ± 0.6 and 3.7 ± 0.8 days, respectively (*P*=0.01).

A potential disadvantage of TPRK is the higher total excimer LASER energy load. In our study, mean ablation time was 140% longer in the TPRK group; however, the Schwind LASER machine tuned so that the majority of the laser energy was delivered to the epithelium, and it was decreased gradually in stromal treatment reaching to its lowest level.

## 4. Conclusion

TPRK and AAPRK produce similar outcome results 3 months postoperatively and after 6 months follow-up. There is shorter surgical time in the TPRK technique than in AAPRK. However, in early postoperative period, there were significant differences in UDVA, pain score, level of haze, and complete corneal epithelial healing time, in which the pain scores were lower, level of haze was less, and healing time was shorter in the TPRK group which resulted in the patient felling that it is a friendly procedure and higher patient comfort in this early postoperative period. Both of procedures are predictable, effective, and safe for correction of myopia and compound myopic astigmatism.

## Figures and Tables

**Table 1:** Demographics and preoperative variables of patients in the TPRK and AAPRK groups. *P* < 0.05 was considered statistically significant. ^*∗*^Statistically significant.

Parameters	Groups		Significance (*P* value)
	TPRK	AAPRK	
MRSE (*D*)	−3.158 ± 1.596	−2.90 ± 1.899	0.089
K readings (*D*)	44.50 ± 1.4	43.6 ± 1.7	0.35
CCT (*µ*m)	490 ± 4.80	496 ± 4.60	0.45
ESR thickness (*µ*m)	340 ± 8	345 ± 7	0.44
Ablation time (sec)	29.71 ± 7.624	12.330 ± 6.138	0.039
Surgical time (sec)	162.17 ± 14.8	243.24 ± 98.7	<0.001^*∗*^
Healing time (days)	3.20 ± 0.686	4.600 ± 1.969	<0.001^*∗*^
Optical zone diameter (mm)	6.734 ± 0.194	6.745 ± 0.201	0.98
Transitional zone diameter (mm)	1.036 ± 0.371	0.995 ± 0.364	0.645
Overall satisfaction (high)	90%	88%	0.46

**Table 2:** Efficacy index is the mean postoperative UDVA/the mean preoperative BCDVA. *P* < 0.05 was considered statistically significant. ^*∗*^Statistically significant.

Efficacy index					
Treatment group	1 day	1 week	3 month	6 months	6 months
TPRK	0.714 ± 0.0786	0.903 ± 0.0683	1.021 ± 0.128	1.036 ± 0.117	1.047 ± 0.116
AAPRK	0.477 ± 0.0872	0.558 ± 0.0564	0.921 ± 0.0789	0.987 ± 0.064	1.020 ± 0.0801
*P* value	<0.001	<0.001^*∗*^	0.20	0.35	0.47

**Table 3:** Safety index is the mean postoperative BCDVA/the mean preoperative BCDVA. *P* < 0.05 was considered statistically significant.

Safety index			
Treatment group	1 month	3 months	6 months
TPRK	1.030 ± 0.122	1.039 ± 0.115	1.047 ± 0.116
AAPRK	0.933 ± 0.0760	0.990 ± 0.0622	1.020 ± 0.0801
*P* value	0.25	0.37	0.47

## Data Availability

The datasets used and/or analyzed during the current study are available from the corresponding author on reasonable request.

## Abbreviations

LASIK: Laser in situ keratomileusis
LASEK: Laser-assisted subepithelial keratectomy
Epi-LASIK: Epithelial laser in situ keratomileusis
PRK: Photorefractive keratectomy
TPRK: Transepithelial photorefractive keratectomy
AAPRK: Alcohol-assisted photorefractive keratectomy
UDVA: Uncorrected distant visual acuity
BCDVA: Best corrected distant visual acuity
MRSE: Manifest refractive spherical equivalent
MMC: Mitomycin C
BSS: Balanced salt solution
QID: Four times a day
TID: Three times a day
BID: Twice a day
CCT: Central corneal thickness.
